# Supplementary material for: Experiences of being treated with autologous haematopoietic stem cell transplantation for aggressive multiple sclerosis: A qualitative interview study
Source: PLoS One. 2024 Feb 7;19(2):e0297573. doi: 10.1371/journal.pone.0297573 (PMC10849216; doi:10.1371/journal.pone.0297573)
Supplement: Attachment — Submitted filename: Exp of AHSCT in MS 231026 Response to reviewers.docx [file pone.0297573.s001.docx]

Dear Dr. Zimmerman,

On behalf of all the authors, I would like to thank you for considering our study for publication in *PLOS ONE*. We also would like to sincerely thank the reviewers for their time and effort in reading our study and providing us valuable comments on how to improve the text. We present our remarks point by point below, with the comments by the reviewers in bold.

Sincerely,

Andreas Tolf

# Journal requirements

**1. Please ensure that your manuscript meets PLOS ONE's style requirements, including those for file naming.**

We have now formatted the headings and table titles according to the attached instructions; these changes have not been marked in the document "Revised Article with Changes Highlighted".

**2. In your Data Availability statement, you have not specified where the minimal data set underlying the results described in your manuscript can be found. PLOS defines a study's minimal data set as the underlying data used to reach the conclusions drawn in the manuscript and any additional data required to replicate the reported study findings in their entirety. […] If there are ethical or legal restrictions to sharing your data publicly, please explain these restrictions in detail.**

The underlying data that forms the basis for the study and its conclusions are the interviews conducted with the participants. These interviews were recorded and then transcribed verbatim. Primarily, it is these transcriptions that have been used for data processing, but also to some extent the audio files and the impression from the actual interview sessions. During the interviews, personal details emerged about relatives, former partners, friends, parents, and children. There are also details that make it probable for acquaintances, friends, and other people in the participants' surroundings to identify the participants based on the fully transcribed interview.

Therefore, out of ethical considerations for the participants' integrity, as well as for the personal integrity of individuals mentioned in the interviews, we have decided not to make the source material available in the form of the audio recordings or the full transcriptions. To compensate for this, we have chosen to extensively quote the participants in the manuscript.

If the Editor or Reviewers so wish, we are happy to share the anonymised transcriptions with them in confidence that the data will not be published for public accessibility.

**3. Please review your reference list to ensure that it is complete and correct.**

We have reviewed the reference list and found no inaccuracies or articles that have been retracted. No changes to the reference list have been made.

# Reviewer 1

**1. Please add EDSS at baseline and the 10-y follow-up in Table 1.**

We have added EDSS at AHSCT and at the time of the interview in Table 1, as suggested.

**2. As few patients who did not observe any improvements are mentioned, I suggest expanding this section, shall there be enough material in the interviews.**

Thank you for this question, which shows that our medical description of the cohort was not sufficient. In fact, all participants improved after the treatment, even if not everyone improved as much as they had hoped for before the treatment. The improvement is reflected, among other things, by the EDSS values, according to the figure below from the following publication, which is referred to in the manuscript:

Tolf A, Fagius J, Carlson K, Akerfeldt T, Granberg T, Larsson EM, Burman, J. Sustained remission in multiple sclerosis after hematopoietic stem cell transplantation. Acta Neurol Scand. 2019;140(5):320-7.

To clarify this, in addition to the EDSS values in Table I, we have added more clinical information in Table I, as also requested by Reviewer II: EDSS before AHSCT, annual relapse rate prior to AHSCT, length of hospitalization, acute side effects related to the treatment, EDSS at the time of the interview, as well as disease activity and any deterioration after AHSCT.

**3. Among cases that continued to worsen despite the procedure, did someone manifest any regrets about the decision to undergo AHSCT?**

None of the participants expressed any regret about having undergone AHSCT. On the contrary, one of the participants, ‘Daniel’, who actually had relapses after the treatment, described how his life, despite this, had returned to normality: ‘After this treatment, my life is back to normal again’. We have clarified in the text that none of the participants regretted the treatment by adding the following section:

*Even though the outcome of the treatment did not always lead to the improvement that was expected, none of the participants expressed regret about having undergone AHSCT.*

**4. Lines 579-580: I suggest rephrasing the sentence “After AHSCT, previously lost functions returned, with recovery of autonomy, identity, and a sense of normality” specifying that such improvement happened in most cases (but not in 100% of the patients treated).**

We have revised the sentence as suggested:

*After AHSCT, previously lost functions returned, with recovery of autonomy, identity, and a sense of normality in most of the participants.*

Reviewer 2 

**1. The sample size of this study is small, it would be more compelling if other centers were approached to contribute qualitative information to ensure that the themes of responses are generalizable.**

We agree that a significant limitation of this study is its small number of participants. By including more participants, the generalisability of the conclusions could have been strengthened.

However, this cohort is almost identical to the first ten patients who underwent this treatment in Sweden. By including all centres that performed AHSCT against relapsing-remitting MS in our country, only one additional patient could have been considered for participation in the study without changing the time interval for inclusion in the study (2004-2007), or by including patients who underwent AHSCT for relapsing-remitting MS in other countries, which would have presented other challenges, mainly for linguistic and logistical reasons.

With the experiences from this study, we look forward to conducting future studies using qualitative methodology on patients who have undergone AHSCT for relapsing-remitting MS and to include more participants.

**2. There are, of course, very different mobilization, conditioning, and graft-processing protocols with AHSCT, and the protocol used in this center was not described. The difference in intensities should be outlined.**

We have added information on the protocols used and the differences in intensity between different protocols in the Methods section:

*AHSCT can be conducted according to various protocols of differing intensities. These are commonly classified as high-, intermediate-, or low-intensity protocols. All ten participants underwent mobilisation of the haematopoietic stem cells with a single dose of cyclophosphamide 2g/m^2^ and filgrastim 5-10 µg/kg/day over 6-7 days, followed by collection of the stem cells on day 10 or 11 after the start of mobilisation. The graft was then cryopreserved without further manipulation. Nine of the participants (all except the youngest, ‘Brandon’) then underwent conditioning with the intermediate intensity BEAM-ATG regimen consisting of carmustine 300 mg/m^2^, etoposide 800 mg/m^2^, cytarabine 800 mg/m^2^, melphalan 140 mg/m^2^, and antithymocyte globulin 10 mg/kg. "Brandon" was treated with a low-intensity conditioning regimen consisting of cyclophosphamide 200 mg/mg and antithymocyte globulin 6 mg/kg.*

**3. In addition, relating participant responses to pre-transplant disease activity, post-transplant disease activity, disability worsening, or side effects such as infection or length of hospitalization would be important to include.**

We share the view that such information can enhance the comprehension of the participants' responses. Therefore, in Table I, we have included the suggested clinical details: EDSS before AHSCT, annual relapse rate prior to AHSCT, length of hospitalization, acute side effects related to the treatment, EDSS at the time of the interview, as well as disease activity and any deterioration after AHSCT.

**4. A quality of life or patient reported outcome questionnaire should be included as well to correlate with findings, there have been multiple studies of AHSCT that has shown a positive effect from Transplant.**

We agree that such a questionnaire could have been included in the results and provided additional perspectives through quantitative data. Unfortunately, a formal assessment of quality of life was not made at the time of the interview. **5. The methodology of conducting the interview is detailed, however the analysis was not clear. Simply stating that “Consensus on the coding, the latent content, and the level of abstraction was reached through exhaustive virtual meetings with the three coding researchers” is insufficient.**

We are grateful for this opinion and have revised the description of the method section to better explain the steps of the analysis:

*Qualitative content analysis was used for the analysis, according to the method described and elaborated by Graneheim, Lundman and Lindgren [19, 22, 23]. The transcribed interviews constituted the units of analysis. After multiple readings to grasp the entirety of the content and to ‘obtain a sense of the whole’ [23], the individual meaning units in the text were identified, representing a ‘constellation of words or statements that relate to the same central meaning’ [19]. In order to improve readability for further analysis, the meaning units were then condensed, transitioning from the verbatim transcription to a clearer, more formal expression, while preserving the original inherent meaning. Subsequently, these statements were examined and interpreted to establish their deeper meaning. This process, and interpretation of the latent content, as outlined above and exemplified in Table 3, was interpreted by three of the researchers independently (GF, HG, AT), using the software Microsoft Word (version 2016).*

*Interpreting the latent meaning is one of the most delicate steps in qualitative content analysis and requires an understanding of the entire context in which an individual "meaning unit" is situated. Often, such interpretation is intuitive and uncomplicated, but sometimes different researchers make different assessments and interpretations. This can involve perceiving a meaning unit differently or having differing views of the context. To strengthen trustworthiness, the analysis was therefore conducted by the three researchers entirely independently, as described above. In joint and exhaustive digital meetings, the individual analyses of the three interpreting researchers were then compared. In-depth discussions were held about important passages and interpretations that might differ, with the aim of achieving consensus on the coding, latent content and level of abstraction. AT kept a record and noted the interpretations of the latent meaning and codes that had emerged from the consensus process. At times, the audio recordings of the interviews were also played to clarify emphases and vocal expressions that could affect the interpretations. Through the joint discussions, a deeper understanding of both the individual statements, the codes, and the whole was also achieved. An example of the process with the different analysis steps is presented in Table 3.*

*From the deliberations in those sessions, GF and HG formulated themes and subthemes. A theme was perceived as a ‘thread of meaning’ [23] connecting latent content across various meaning units and codes, reflecting the text's latent content. The themes can further be subdivided into subthemes. [19] AT performed the re-contextualisation [23] by grouping the codes with their respective meaning units into the formulated themes and subthemes, using the software MAXQDA 2020 (version 20.4.2). Based on this compilation, representative citations were chosen to illustrate the themes and sub-themes (AT). Citations were translated from Swedish to English by an authorized translator with experience of translating both fictional and scientific literature, and nuances in the translations were discussed in sessions with the translator. Consensus on the final themes, subthemes, and appropriate citations was reached* in a meeting with all authors.

**6. There are software programs such as NVIVO for qualitative data analysis, as well as criteria for evaluating qualitative studies and data that should be employed. This may allow for figures to be generated and help the article be visually appealing.**

We agree with the observation that the article is notably dense in textual content. During the re-contextualisation after the analysis, we used the software MAXQDA 2020 to structure the quotations. However, this software did not offer a satisfactory or clarifying graphical representation of our material.

**7. At minimum, EQUATOR network reporting guidelines for qualitative studies should be included.**

In the reporting of this study, we have used the two recommended EQUATOR network reporting guidelines, the COREQ checklist and the SRQR guidelines, as reported in the first paragraph in the Methods section.

**8. The article should also be proofread and edited in formal English.**

In this study, in accordance with the practice for qualitative studies, we have chosen to present the participants' statements verbatim, without formalizing the language, except that the quotes have been translated by a certified translator from Swedish to English. The aim of presenting the participants’ statements verbatim has been to enhance credibility and transparency. The text that is not quotes is written in formal English and has undergone proof-reading by a certified translator. We would still like to apologize for any linguistic or grammatical shortcomings, which we have endeavoured to identify and rectify.

**9. There are different fonts used in this PDF which may be a small oversight but should be corrected.**

The text has now been formatted to a consistent font.
